# Premature Symptomatic Alopecia

**Published:** 1885-07

**Authors:** J. A. Abney

**Affiliations:** Lufkin, Texas


### PREMATURE SYMPTOMATIC ALOPECIA.

*By J. A. Abney, M. D., Lufkin, Texas.*

For Daniel's Texas Medical Journal.

E. M., a female eight years old, complexion blonde, of nervous, sanguine temperament, strumous diathesis, mother delicate, (maternal grand mother consumptive from repeated attacks of pneumonia, not hereditary, at least not traceable), family history otherwise good; has suffered since three years of age with hypertrophy of tonsils, the result, apparently, of the continued irritation produced by an attack of rubeola immediately succeeded by pertussis, the hypertrophied glands in turn kept up more or less irritation of the mucous surface of the nares and eyelids, for which she had been treated alternately with syrup of iodide of iron, iodide of potassium, bi chloride of mercury, tincture of iodine, tincture of iron, etc., etc., with the various topical applications advised under such circumstances, without any permanent relief.

While attending a school in May, 1884, in which were two little girls affected with tinea tonsurans, she was suddenly attacked with a falling of the hair from the scalp in patches, accompanied with itching and bran-like scales. I at once diagnosed tinea tonsurans, and began an active course of treatment accordingly, with strongly corbolized oil, citrine ointment, etc., externally, and Fowler's solution internally. I soon succeeded in getting up a fine state of irritation in the bald spots on the scalp, but to my astonishment and chagrin, the spots kept enlarging, until something near one-third of the scalp was involved; in the meantime, the irritation of the eyelids and nares had given way. Despairing of success, I applied to Dr. F. E. Daniel, at Austin,for help, in December last. He at once wrote me he thought I had a case of symptomatic Alopecia, a reflex of the diseased mucous membrane, and advised a cessation of irritant applications, and the use of emolient tonics, zinc oleate, etc., to relieve inflammation; and for constitutional treatment, recommended cod liver oil with the hypophosphites of lime and soda. The advice was followed, and in one month's time I had the pleasure of seeing the bald spots nicely cleaned off, the inflammation having subsided, and a nice coat of new hair started. I continued treatment about three months. At this writing (June) patient has a fine, full coat of hair, her skin is clear and bright, and she is in the enjoyment of perfect health, except the hypertrophy of tonsils, which still exists, though the irritation of eyes, and posterior nares has not returned.

The points of interest in the case as it occurs to me are: First, the incorrect diagnosis and the grounds which led to it; second, the necessity of always arriving at a diagnosis by strict rules of differentiation, by exclusion; and thirdly, we are reminded of the fact that in cod liver oil, with the hypophosphites of lime and soda, we possess a remedy that is very potent for good, not only in symptomatic Alopecia, but in all cases of impaired nutrition, especially where we wish to renew the hair or osseous system. Hoping some professional brother may be benefited by the report of this case, it is respectfully submitted.

